# Epidemiology of Tumor-Induced Osteomalacia in Germany Based on Real World Data

**DOI:** 10.1007/s00223-023-01148-2

**Published:** 2023-11-18

**Authors:** Melanie May, Ralf Oheim, Leonore Bovy, Axel Doess, Dirk Maessen, Benno Neukirch, Raeleesha Norris, Angela Williams, Bo Abrahamsen

**Affiliations:** 1 Department of Payer Value & Patient Access, Kyowa Kirin GmbH, Monschauer Str. 1, 40549 Duesseldorf, Germany; 2 Department of Osteology and Biomechanics, University Hospital Hamburg-Eppendorf (UKE), Lottestraße 59, 22529 Hamburg, Germany; 3 InGef - Institute for Applied Health Research Berlin GmbH, InGef, Otto-Ostrowski-Straße 5, 10249 Berlin, Germany; 4 Department of Franchise Nephrology, Kyowa Kirin GmbH, Monschauer Str. 1, 40549 Duesseldorf, Germany; 5 Hochschule Niederrhein, University of Applied Sciences, Competence Center Routine Data, Reinarzstraße 49, 47805 Krefeld, Germany; 6 Department of HEOR, Kyowa Kirin International Ltd, 2 Globeside, Fieldhouse Lane, Marlow, SL7 1HZ UK; 7 Department of Medicine 1, Holbæk Hospital, Smedelundsgade 60, 4300 Holbæk, Denmark; 8 Department of Clinical Research, University of Southern Denmark (SDU), Campusvej 55, 5230 Odense, Denmark

**Keywords:** TIO, Epidemiology, SHI claims data analysis, Real-world data, FGF-23

## Abstract

Tumor-induced osteomalacia (TIO) is an ultra-rare disease caused mostly by benign tumors that secrete fibroblast growth factor-23. Because of nonspecific symptoms, the diagnostic delay is long, and therapy can be challenging. Moreover, epidemiological data on TIO are scarce owing to its rarity. Therefore, this study aimed to quantify TIO’s incidence rates and prevalence in Germany. Retrospective longitudinal and cross-sectional analyses were conducted using anonymized German claims data from the statutory health insurance (SHI) database. This database, which comprises the data of approximately 5 million insurants, is a representative sample of the German population and supports national projections. As there is no unique International Statistical Classification of Diseases and Related Health Problems (ICD) code for TIO, operational categories based on different surrogates were defined to determine the prevalence and incidence rates of TIO among probable patients. This study showed that TIO has a prevalence of (documented code, advanced imaging, medication, or tumor removal) 0.187 per 100,000 persons and an incidence rate of ≤ 0.094 per 100,000 person years. This analysis provides the first epidemiological insight into German patients with TIO. Despite the general limitations associated with the analysis of SHI claims data of ultra-rare diseases, we believe that this analysis provides a sound basis for further analysis, particularly with regard to the care situation of patients with TIO.

**Supplementary Information:**

The online version contains supplementary material available at 10.1007/s00223-023-01148-2.

## Introduction

Tumor-induced osteomalacia (TIO) is a rare paraneoplastic syndrome characterized by severe hypophosphatemia and osteomalacia with potentially serious sequelae and disability [1]. TIO is caused by a phosphaturic mesenchymal tumor (PMT), mostly benign, that expresses and secretes fibroblast growth factor-23 (FGF23), or in rare cases, other phosphatonins, in increased amounts. The causative tumors, which are highly heterogeneous, can be localized in any anatomical region; however, they are predominantly found in the (lower) extremities and can occur in the bone, soft tissue, or skin [1, 2].

FGF23 plays a central role in regulating phosphate metabolism. In the proximal renal tubules, FGF23 accumulation downregulates the expression of the type II sodium-phosphate cotransporter system, which is involved in the reabsorption of phosphate from the primary urine. Consequently, increasing amounts of phosphate are excreted by the kidneys, leading to the development of severe hypophosphatemia. In addition, FGF23 represses calcitriol activation by inhibiting the transcription of the enzyme, 1-α-hydroxylase (CYP27B1) and stimulating the expression of 24-α-hydroxylase (CYP24A1), thereby promoting calcitriol catabolism. Together, these mechanisms contribute to the reduced intestinal absorption of phosphate and calcium, which are vital for unimpaired bone mineralization [2–5].

TIO is primarily a disease in adults; however, it can occur in children in rare cases [6, 7]. In children, chronic hypophosphatemia due to renal phosphate loss manifests as pronounced rickets with severe deformities, such as knock knees or bowed legs, leading to gait abnormalities and short stature [6]. In adulthood, eponymous osteomalacia is predominant and can result in the development of bone pain and (pseudo)fractures, skeletal deformities, mineralizing enthesopathy, and loss of height. TIO typical symptoms include muscle and bone pain, fatigue, and muscle weakness [2, 8, 9] Owing to this nonspecific symptomatology, the diagnosis of TIO is often made months or years after the onset of the first symptom [7, 10]. Moreover, owing to its complex and severe clinical picture, patients with TIO have severely impaired mobility, quality of life, and participation in an active life [1, 9, 11].

The only available curative treatment for TIO is complete tumor resection. A few days following resection, FGF23 and phosphate concentrations can be normalized, leading to improved clinical symptoms [1]. In addition, radiation or tumor ablation procedures should be considered for further interventions [12–15]. However, in some cases, the tumor cannot be resected completely or localized. To date, no targeted drug therapy has been available for patients with TIO. Because of the disease’s rarity and lack of alternative therapeutic options, no targeted pharmacotherapies, guidelines, or consistent therapeutic recommendations exist for TIO treatment. Consequently, TIO treatment is limited to replacement therapy using oral phosphate and vitamin D as described in previous case reports and case series [10, 11, 16]; however, its efficacy has never been confirmed or quantified in robust clinical studies*.* As a new treatment option, burosumab is available as a fully human monoclonal antibody (AK, IgG1) that specifically binds to the phosphaturic factor FGF23 in adults and children with TIO associated with phosphaturic mesenchymal tumors that cannot be curatively resected or localized [16–19].

Owing to the disease’s rarity and the lack of an ICD-10 code, epidemiological data on TIO are scarce. Two epidemiological studies examined the prevalence and incidence of TIO: First, a Japanese study did not provide precise TIO prevalence rates to allow suggesting epidemiological statements for TIO. However, more recently a found a contact prevalence of 0.47 per 100,000 for the total population and 0.33 per 100,000 pop if analyses were restricted to adults [20, 21]. The European medicines agency (EMA) reports TIO prevalence and incidence [orphan drug designation (ODD)] rates of 0.09 and 0.13 per 100,000 individuals, respectively. However, this is subject to uncertainty, as the primary source of the ODD report is unclear [22].

Hence, in this study, we aimed to determine the administrative prevalence and incidence rates of TIO using claims data from the German statutory health insurance (SHI) database [23], raise awareness about this rare disease, and educate practitioners and health authorities regarding TIO coding challenges. By using additional clinical information that was not available in the Danish epidemiology study, we further refined the operational definition of TIO in claims data.

## Materials and Methods

### Data Collection and Study Design

The anonymized SHI claims dataset (research database of the InGef Institute for Applied Health Research Berlin GmbH), containing information from approximately 9 million insurants, was used in this study. A sample size of approximately 4.7 million individuals was drawn annually, and adjusted for age and sex based on national statistics. This sample is considered representative of the entire German population and supports national projections [24]. The SHI claims data were analyzed according to the guidelines of the "Good Practice for Secondary Data Analysis (GPS) and the STROSA checklist [25, 26].

In addition to demographic information, data records included anonymized core data on the insurant and healthcare providers, billing information on utilized inpatient and outpatient health services, and prescribed pharmaceuticals (ICD-10-GM, OPS, EBM, G-DRG, ATC) [27–29].

To ensure that only recent information was used, this retrospective analysis was restricted to a period of the most recent 6 calendar years [30]. For data privacy and regulatory reasons, no subgroups containing fewer than five persons were analyzed. All subject-level and provider-level data in the InGef research database were anonymized to comply with the German data protection regulations and federal law.

**Fig. 1:**
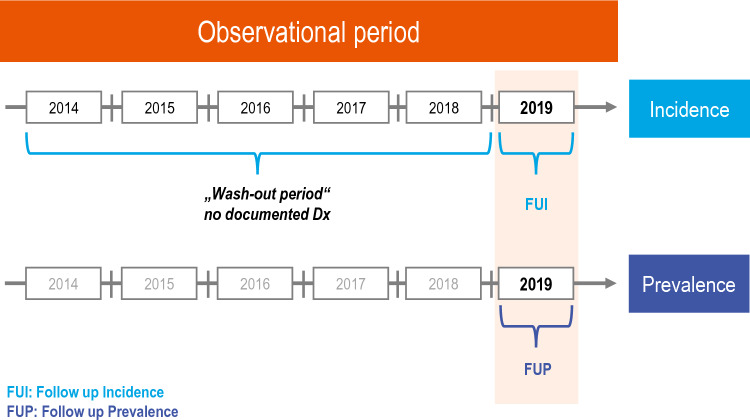
Observational period and study design

As a retrospective cohort study, cross-sectional and longitudinal analyses of (administrative) TIO prevalence and incidence were conducted for the year 2019 (Fig. 1). The observation period was limited to 2019 to avoid possible distortions and underestimation owing to the Covid 19 pandemic-related lower utilization of healthcare services in 2020, especially when considering, as is the case here, a very rare disease [31–34].

### Study Population

The Danish registry study by Abrahamsen et al. [21] and a German publication on TIO localization and therapy [7] were used as the initial template for this study’s design though additional refinements were added. Patients of all ages and sexes were included in this analysis using the ICD-10-GM classification (Table 1). According to orphanet, oncogenic or tumor-induced osteomalacia is recommended to be coded as M83.8 (*Other osteomalacia in adults)* [35]. As M83.8 only includes adult patients, the codes E83.31 (*Vitamin D-dependent rickets)* and E83.39 (*Disorders of phosphate metabolism and phosphatase, unspecified)* were used to capture presumed patients with TIO aged < 18 years. Patients’ data were included in the cohort based on the presence of at least one of the above-mentioned ICD codes documented as a confirmed outpatient diagnosis in at least two-quarters (M2Q criterion) or as an inpatient main or secondary diagnosis. In 2019, the identified incident cases did not have any of the predefined ICD-10-GM diagnoses in the previous years (2014–2018) (Fig. 1). Patients with hypophosphatemic osteomalacia due to other causes, such as drug therapies, or related disorders were excluded (Table 1). As in the Danish study, patients on iron infusions, which can lead to ferric carboxymaltose, multiple myeloma, Fanconi syndrome, familial hypophosphatemic rickets (XLH), or chronic kidney diseases were excluded [6, 21].

**Table 1:** Inclusion and exclusion criteria

Category	Code	Description
*Inclusion criteria*		
ICD-10-GM	E83.3	E83.31 vitamin D-dependent rickets
		E83.39 Disorders of phosphorus metabolism and phosphatase, unspecified
ICD-10-GM	M83.8	other osteomalacia in adults
*Exclusion criteria*		
ICD-10-GM	B20–B24	HIV disease
	C90.0	multiple myeloma
	E20	Hypoparathyroidism
	E83.30	familial hypophosphatemic rickets (XLH and others)
	E83.38	other disorders of phosphorus metabolism and phosphatase (Fanconi syndrome)
	N17–N19, N25	chronic kidney disease (CKD)
ATC	B03AC	iron, parenteral preparations (iron infusion)
ATC	M05BX05	burosumab injection*
OPS	6-00b.4	

*as an indicator for XLH, as it was not approved for TIO at that time

Deviating from the Danish study, patients with the human immunodeficiency virus (HIV) infection, on tenofovir medication, patients with hypoparathyroidism and patients with Burosumab injection were also excluded [6].

### Case Definition

Because of the unspecificity of the TIO code and ambiguity in the identification of patients with TIO, further surrogates were used to determine the patient population using the code M83.8. The overall surrogates were based on established clinical guidelines [36] and the Danish registry study [21]. Five subgroups were formed and optimized for the German healthcare system:

- A(*Possible* TIO): Fulfillment of the inclusion criteria (ICD-10-GM coding).
- B(*Possible* TIO with advanced imaging): Fulfillment of the inclusion criteria and at least one advanced imaging procedure.
- C(*Probable* TIO) (medication or tumor removal): Fulfillment of the inclusion criteria and at least one prescription for a specific medication or one procedure for tumor removal.
- Group B + C: Fulfillment of the inclusion criteria and at least one advanced imaging procedure, one procedure for tumor removal, or one prescription for a specific medication.
- D(*Definitive* TIO): at least one advanced imaging procedure and one tumor removal procedure or one prescription for a specific medication.

Advanced imaging and tumor removal procedures were defined using OPS or EBM codes. The outpatient prescriptions for the defined TIO-specific medication were categorized according to the ATC classification (Table 2 and Supplementary Table S1). Unlike the Danish study, which was able to capture only one-alpha hydroxylated vitamin D as a TIO relevant medication, we were able to include phosphate and calcium.

**Table 2:** Definition of the surrogates

Surrogate	Definition
Advanced imaging	PET, PET/CT, SPECT, SPECT/CT, EBT, scintigraphy
Medication	alfacalcidol, calcitriol, phosphate, calcium
Tumor removal	surgical tumor removal, radiotherapy
Laboratory intervention	Calcium, Phosphate, AP, Gamma GT, CRP, TSH, Osteocalcin PTH, DPD, FGF23, VitD
Genetic testing	Gene mutation analysis, molecular genetic diagnostics

In contrast to the Danish study, additional information about patient characteristics and healthcare resource use (HCRU), laboratory, and genetic tests were used as descriptive outcome parameters; however, they were not used for classifying the subgroups. Laboratory tests focused on calcium phosphate metabolism, bone turnover markers, and screening parameters for secondary osteoporosis. Genetic tests were defined as gene expression analysis, genetic testing, and molecular genetic diagnostics (Supplementary Table S1). In addition, a combination of the diagnoses associated with the TIO disease (such as bone pain), sick leave, prescriptions, and average defined daily doses (calculated DDD) of analgesics was defined as indicators of the disease burden. As the causative tumors are mainly benign PMT, which are predominantly localized in the extremities and can occur in the bone, soft tissue, or skin [1, 2, 7], the number of patients with additional coded benign tumors (ICD-10-GM, D10–D26) were calculated to build the subgroups (Supplementary Table S1). The projections for the German population were based on the Destasis data for the year 2019, with 83,166,700 inhabitants [37]. For data protection, only aggregated data were reported, and patient numbers from one to four could only be marked with < 5. To calculate the rates in these cases, < 5 was replaced with “4.” Data management and statistical analyses were performed using Microsoft R Open 4.0.2 [38].

## Results

### Prevalent TIO Patients 2019

**Fig. 2:**
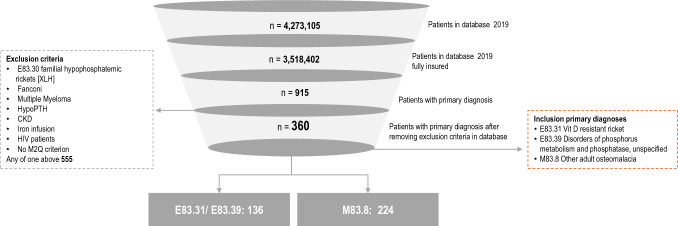
Patient flow and inclusion and exclusion criteria of prevalent patients 2019

**Fig. 3:**
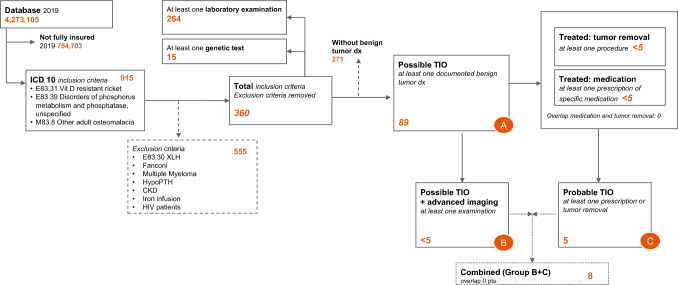
TIO subgroups

After the exclusion criteria were applied, 360 patients that fulfilled the inclusion criteria were identified in the database in the year 2019. Approx. 60% of the patients had M83.8 as unspecific adult osteomalacia code (Fig. 2). Of these, 89 showed at least one documented benign tumor diagnosis (group A). Of these, fewer than five patients were assigned to group B (advanced imaging) and five patients to group C (medication or tumor removal). None of the patients fulfilled the criteria for group D (definitive TIO with advanced imaging and prescription or tumor removal). After excluding overlaps between groups B and C, eight patients in the combined group were analyzed Approx. 70% of the patients received a specific laboratory examination (264). 15 of 360 (4%)patients had at least one genetic test as defined above (Fig. 3).

### Incidence, Prevalence, and National Projection

In 2019, 78 incident patients who met the inclusion criteria were identified. Of these, 13 showed the presence of at least one documented benign tumor diagnosis (group A). The 13 patients had an average age of 59.6 years (11.6; 34–85) with 61.5% women. The ICD distribution in the group total and group A was identical (approx. 50:50). Fewer than five patients died within 1 year of the observation period. An incidence rate of 0.304 per 100,000 person years was calculated for group A (possible TIO). For the other groups, an incidence rate ≤ 0.094 per 100,000 person years (< 5) was calculated.

The prevalence in 2019 was between 0.070 and 2.083 per 100,000 persons. Group B + C, which fulfilled at least one of the defined TIO criterion, showed a prevalence of 0.187 per 100,000 persons. When the results were extrapolated to the German population, there were 156 prevalent and between 19 and 78 incident patients in Groups B + C in 2019 (Table 3).

**Table 3:** Overview of the prevalence and incidence rates for 2019 and national projections for the German population

	Study data		National projection for Germany	
	Prevalence 2019 per 100,000 pop	Incidence rate 2019 per 100,000 p.y	Prevalent patients 2019	Incident patients 2019
A possible TIO	2.083	0.304	1,732	253
B Possible TIO and advanced imaging	0.070	≤ 0.094	58	19–78*
C Probable TIO (medication or tumor removal)	0.117	≤ 0.094	97	19–78*
B + C (imaging or medication or tumor removal)	0.187	≤ 0.094	156	19–78*

*Ranges are the result of < 5; < 5 can be replaced with “1” as minimum and “4” as maximum

### Patient Characteristics Prevalent TIO Patients 2019

Most of the 360 patients were women (77.5%) with an average age of 58.6 (20.4; 0–91) years. Approximately 60% of the patients were coded as M83.8. In group A (possible TIO, 89), two patients (2.2%) were aged < 18 years. The average age was 60.2 (15.7; 4–85) years with a sex distribution of 24.7% men and 75.3% women. Owing to their small numbers (< 5), no details regarding patient characteristics for group B were provided. The average age of the five patients in group C was 59.2 (10.4; 44–69) years. Group B + C with eight patients had the oldest patients with an average age of 62.5 (11.7; 44–87) years. There were no patients aged < 18 years in groups B–D. The mortality rate was 1.4% in 2019 for all patients in group A. In the other groups, no patient died within 1 year. Most of the patients were assigned the code M83.8 with approximately 70% in group A (Table 4).

**Table 4:** Demographical characteristics of patients with prevalent TIO in 2019 for each group

Case definition	Total	A possible TIO	B Possible TIO and advanced imaging	C Probable TIO medication or tumor removal	B + C imaging or medication or tumor removal
N	360	89	< 5	5	8
Average age (SD; Range)	58.6 (20.4; 0‒91)	60.2 (15.7; 4‒85)	< 5	59.2 (10.4; 44‒69)	62.5 (11.7; 44‒84)
< 18	24 (6.7%)	2 (2.2%)	–	–	–
Men	81 (22.5%)	22 (24.7%)	< 5	< 5	< 5
Women	279 (77.5%)	67 (75.2%)	< 5	< 5	< 5
Mortality	5 (1.4%)	< 5	–	–	
Composition of ICD-10 groups					
E83.3 (E83.31 or E33.39)	136 (37.8%)	29 (32.6%)	< 5	< 5	< 5
M83.8	224 (62.2%)	60 (67.4%)	< 5	< 5	5 (62.5%)

**Fig. 4:**
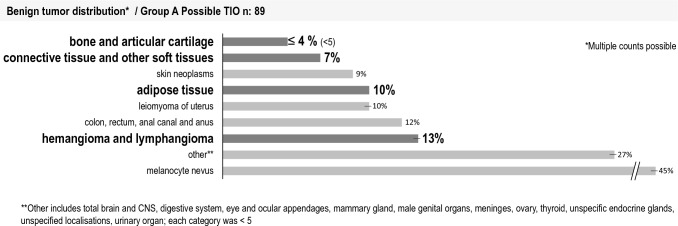
Distribution of benign tumor diagnoses by documented ICD-10-GM, 2019 for group A possible TIO, n: 89

The benign tumor distribution (Fig. 4) showed that the mesenchymal tumors that could cause TIO (marked in bold) were identified in the study population. 13% of the patients had at least one documented diagnosis of hemangioma and lymphangioma, followed by tumors in the adipose tissue (10%) and connective and other soft tissues (7%) and < 5 of the patients had bone and articular cartilage tumors.

### Individual Patient Impact (Burden of the Disease)

Almost every patient had at least one defined diagnosis, indicating pain or restrictions in quality of life. Up to 80% of the patients had at least one sick leave day within one year, with an average sick leave between 40 and 300 days within a year. More than every second patient received at least one prescription for analgesia, with an average DDD of approx. 20 (Table 5).

**Table 5:** Overview of individual disease burden for prevalent patients in 2019 for each group

Case definition	Total	A Possible TIO	B Possible TIO and advanced imaging	C Probable TIO medication or tumor removal	B + C imaging or medication or tumor removal
N	360	89	< 5	5	8
Any of one defined dx*	77.5%	78.7%	< 5	< 5	87.5%
At least one day sick leave in FU total**	19.0%	76.0%	< 5	< 5	< 5
Average number of annual sick leave days**	47.1	45.1	153.0	293.0	223.0
At least one prescription of analgesia ATC N02/M01	61.7%	59.6%	< 5	< 5	62.5%
Average DDD analgesia (SD; Median)	24.4 (24.3; 16.7)	25.9 (28.5; 16.7)	< 5	< 5	15.3 (7.3; 14.9)
Any of one above (diagnoses, sick leave, analgesia)	89.2%	88.8%	< 5	< 5	88%

*Bone pain, back pain, (pseudo-)fracture, muscle weakness, thorac deformity, tooth loss, lumbar disc herniation, spondylarthritis, osteoporosis, myalgia, fibromyalgia, cancer of unknown primary (CUP), fatigue

**Calculation of sick leave share based on employees / those with valid sick leave data (Krankengeldbberechtigte Mitglieder”)

## Discussion

The findings of this study, which is based on the assessment of representative sample data of the German population, in the year 2019, showed that two per 100,000 adults fulfilled the criteria of diagnosis codes that could represent TIO, with 0.187 per 100,000 meeting more stringent operational criteria, including medications, advanced imaging, or tumor removal. Accurate identification of TIO cases in epidemiological studies is challenging because of the lack of a specific ICD-10 code. However, compared with the scarce data on TIO prevalence and incidence rates, our results fit within the expected ranges [20, 21]. In contrast to prior studies, we were able to focus on patients with a benign tumor diagnosis and we demonstrated good comparability with previously published epidemiological figures in the range of 0.1–0.47 per 100,000 and confirmed, as expected, a low number of cases. When we focused on group B + C, which according to our perspective, is probably the most realistic TIO population because it fulfilled at least one of the criteria of imaging, medication, or tumor removal, the 1 year prevalence and incidence rate (2019) were 0.187 per 100,000 persons and < 0.094 per 100,000 person years. If we had focused on patients with mesenchymal tumors, which can be, depending on the group, approx. 65% of the whole benign tumor population in the study, the range would have been even lower. However, the challenge in using PMT data is that a TIO diagnosis is often made many months before the causative tumor has been identified, removed, and classified as benign.

The patient characteristics are partly comparable with descriptions from available publications, insofar as this can be said for ultra-rare diseases with extremely low numbers of patients. The age distribution in the sixth decade of life reflects the expected ranges based on several case reports [10, 16, 21]. The age distribution of men in this study, [average age of 47.7 years (SD 25.5)] was consistent with other reports (Rendina et al.: 45.0; 39.0–54.0); however, in our study’s population, women were significantly older [average age of 57.2 years (SD 25.5)] than the women in the study by Rendina et al. [average age of 44.0 years (39.0–56.0)] [16]. In our analysis, there was a higher proportion of women to men (78:22), which was consistent with the Danish registry study with a women proportion of 60% [21]; however, the recent report by Rendina et al. states that, in contrast to the previous study, TIO occurs preferentially in adult men (55:45). Similar results were reported by Bosman et al., with 60% male population [10, 16]. These differences most likely result from the different ways in which the data were collected rather than a true difference in sex predilection between countries. The patients’ high burden of disease can be shown using TIO-associated comorbidities and clinical consequences. Patients suffered from bone and back pains, osteoporosis, and (pseudo-) fractures. The importance of pain has also been demonstrated in recent publications [16]. Up to 80% of the patients had at least one sick leave day, with an average of 40 to 300 sick leave days. In comparison, published sick leave days for the German population on average are 10.9 sick leave days per year, showing that TIO patients on sick leave took a longer time to recover [38]. More than 50% of patients had at least one prescription of analgesia, with an average daily dose of approx. 20 DDD per patient. Compared to the published analgesia doses of the German population (average of 2.6–8.8 DDD (N02/M01) per year, independent of age, sex, and morbidity) [39], our results showed that the DDD for TIO patients is clearly above this, and this factor should therefore be considered more closely in further analyses.

Strength and limitations of the SHI claims data analysis: Our findings must be interpreted in line with some limitations inherent to claims data. First, there was no specific code for TIO or the presence of a phosphaturic mesenchymal tumor (PMT). M83.8 is defined as *other osteomalacia in adults* and includes more than oncogenic osteomalacia or TIO. In patients aged  < 18 years, other codes must be used, which, in combination with the defined criteria, could indicate TIO. Accordingly, as with all rare diseases that lack specific coding, operational assumptions must be made. Second, the definition of tumor removal was a combination of predefined procedural codes (Supplemental material S1), which, according to expert opinion, could indicate the removal of a PMT, and an explorative analysis using rankings was made. It is possible that not all the tumor removal codes were identified. Moreover, information regarding successful tumor removal was not available in the claims data. The prescriptions of tumor-specific medications and laboratory examinations were documented only in the outpatient sector. If patients received low-price medication and laboratory examinations in an inpatient setting, the costs (lump sum) were included in the DRG. Furthermore, the results of the laboratory examinations could not be retrieved from the available administrative sources. Also, special tests such as FGF23 or 1,25-(OH)_2_D, though highly informative for TIO work-up, may not be accessible for every clinician seeing patients with hypophosphatemia. Within our observational period, advanced imaging was required, which in some cases needed approval from the insurance company or an individual funding request. Therefore, the number of patients who underwent these examinations might have been underestimated. Despite these limitations, this analysis had enormous strengths, and healthcare research studies with SHI claims data have a high level of acceptance in the healthcare system. The strength of the dataset results from the robustness of the data; hence, it is representative of the German population. This is supported by the results of patient characteristics and epidemiological key figures, which showed consistent results with the literature and expected ranges, despite a higher proportion of women. Approx. 70% of the patients (group A) received a specific laboratory examination and only 4% of the patients had at least one defined genetic test. These results also underpin robustness, because not each patient receives these specific examinations and test, therefore a further division into the defined subgroups was necessary.

The Danish study fortunately provided us with a template and shows that both claims and register data show a good comparability for both European countries. Adjusted for the German healthcare sector and the available dataset, we gain more insights into patient characteristics and were able to further improve subgroup identification through more available specific definable surrogates.

The challenges facing prescribers regarding the suboptimal coding system will probably end with the beginning of the obligatory coding system, *alpha ID for orphans* [41], in 2023 for the inpatient sector in Germany. The outpatient sector will follow at some point. In this study, epidemiological insights on German patients with TIO were obtained for the first time. We would very much welcome international efforts to facilitate the coding of TIO as a specific disease using other coding systems. It is recommended that this work be updated at regular intervals despite possible COVID-19 pandemic bias. In addition, it would be desirable to conduct more patients` care research studies that will provide deeper insights into the care reality of these patients. It is important to increase awareness of this rare disease to decrease its diagnostic delay and, hence, the loss of quality of life of patients with TIO.

### Supplementary Information

Below is the link to the electronic supplementary material.

Supplementary file1 (DOCX 36 KB)

## Data Availability

The data used in this study cannot be made available in the manuscript, supplemental files, or in a public repository due to German data protection laws (Bundesdatenschutzgesetz). To facilitate the replication of the results, anonymized data used in this study were stored on a secure drive at InGef GmbH. Access to the data used in this study can only be provided to external parties under the conditions of the cooperation contract of this research project and can be assessed upon request after written approval (info@ingef.de), if required.
