# Supplementary material for: Epidemiology of Tumor-Induced Osteomalacia in Germany Based on Real World Data
Source: Calcif Tissue Int. 2023 Nov 18;113(6):630–9. doi: 10.1007/s00223-023-01148-2 (PMC10673975; doi:10.1007/s00223-023-01148-2)
Supplement: Supplementary file 1 — Supplementary file1 (DOCX 36 KB) [file 223_2023_1148_MOESM1_ESM.docx]

**Supplemental Table S1** Code list / definitions

| **Label category** | **Description** | **Code_label** | **Code** |
| --- | --- | --- | --- |
| primary_dx | other adult osteomalacia | ICD | M838 |
| primary_dx | vitamin D-resistant rickets | ICD | E8331 |
| primary_dx | disorders of phosphorus metabolism and phosphatase, unspecified | ICD | E8339 |
| secondary_dx | bone pain/ unspecific pain | ICD | R52 |
| secondary_dx | back pain | ICD | M54 |
| secondary_dx | muscle weakness | ICD | M625 |
| secondary_dx | thoracic deformity | ICD | E643 |
| secondary_dx | tooth loss | ICD | K08 |
| secondary_dx | lumbar disc herniation | ICD | M50 |
| secondary_dx | lumbar disc herniation | ICD | M51 |
| secondary_dx | lumbar disc herniation | ICD | M53 |
| secondary_dx | spondylarthritis | ICD | M45 |
| secondary_dx | osteoporosis | ICD | M80 |
| secondary_dx | osteoporosis | ICD | M81 |
| secondary_dx | myalgia | ICD | M791 |
| secondary_dx | fibromyalgia | ICD | M797 |
| secondary_dx | CUP | ICD | C80 |
| secondary_dx | fatigue | ICD | G933 |
| secondary_dx | fracture | ICD | T02 |
| secondary_dx | fracture | ICD | T08 |
| secondary_dx | fracture | ICD | T10 |
| secondary_dx | fracture | ICD | T12 |
| secondary_dx | fracture | ICD | T142 |
| secondary_dx | fracture | ICD | S02 |
| secondary_dx | fracture | ICD | S12 |
| secondary_dx | fracture | ICD | S22 |
| secondary_dx | fracture | ICD | S32 |
| secondary_dx | fracture | ICD | S42 |
| secondary_dx | fracture | ICD | S52 |
| secondary_dx | fracture | ICD | S62 |
| secondary_dx | fracture | ICD | S72 |
| secondary_dx | fracture | ICD | S82 |
| secondary_dx | fracture | ICD | S92 |
| Exclusion_criteria | familial hypophosphatemic rickets | ICD | E8330 |
| Exclusion_criteria | Fanconi syndrome | ICD | E8338 |
| Exclusion_criteria | multiple myeloma | ICD | C900 |
| Exclusion_criteria | hypoparathyroidism | ICD | E20 |
| Exclusion_criteria | acute renal failure | ICD | N17 |
| Exclusion_criteria | chronic kidney disease | ICD | N18 |
| Exclusion_criteria | renal insufficiency not specified | ICD | N19 |
| Exclusion_criteria | diseases due to damage to tubular kidney function | ICD | N25 |
| Exclusion_criteria | HIV | ICD | B20 |
| Exclusion_criteria | HIV | ICD | B21 |
| Exclusion_criteria | HIV | ICD | B22 |
| Exclusion_criteria | HIV | ICD | B23 |
| Exclusion_criteria | HIV | ICD | B24 |
| Exclusion_criteria | iron infusion | ATC | B03AC |
| Exclusion_criteria | burosumab | ATC | M05BX05 |
| Exclusion_criteria | burosumab | OPS | 600b4 |
| tumor_dx | in situ neoplasms | ICD | D00 |
| tumor_dx | in situ neoplasms | ICD | D01 |
| tumor_dx | in situ neoplasms | ICD | D02 |
| tumor_dx | in situ neoplasms | ICD | D03 |
| tumor_dx | in situ neoplasms | ICD | D04 |
| tumor_dx | in situ neoplasms | ICD | D05 |
| tumor_dx | in situ neoplasms | ICD | D06 |
| tumor_dx | in situ neoplasms | ICD | D07 |
| tumor_dx | in situ neoplasms | ICD | D08 |
| tumor_dx | in situ neoplasms | ICD | D09 |
| tumor_dx | benign neoplasm | ICD | D10 |
| tumor_dx | benign neoplasm | ICD | D11 |
| tumor_dx | benign neoplasm | ICD | D12 |
| tumor_dx | benign neoplasm | ICD | D13 |
| tumor_dx | benign neoplasm | ICD | D14 |
| tumor_dx | benign neoplasm | ICD | D15 |
| tumor_dx | benign neoplasm | ICD | D16 |
| tumor_dx | benign neoplasm | ICD | D17 |
| tumor_dx | benign neoplasm | ICD | D18 |
| tumor_dx | benign neoplasm | ICD | D19 |
| tumor_dx | benign neoplasm | ICD | D20 |
| tumor_dx | benign neoplasm | ICD | D21 |
| tumor_dx | benign neoplasm | ICD | D22 |
| tumor_dx | benign neoplasm | ICD | D23 |
| tumor_dx | benign neoplasm | ICD | D24 |
| tumor_dx | benign neoplasm | ICD | D25 |
| tumor_dx | benign neoplasm | ICD | D26 |
| tumor_dx | benign neoplasm | ICD | D27 |
| tumor_dx | benign neoplasm | ICD | D28 |
| tumor_dx | benign neoplasm | ICD | D29 |
| tumor_dx | benign neoplasm | ICD | D30 |
| tumor_dx | benign neoplasm | ICD | D31 |
| tumor_dx | benign neoplasm | ICD | D32 |
| tumor_dx | benign neoplasm | ICD | D33 |
| tumor_dx | benign neoplasm | ICD | D34 |
| tumor_dx | benign neoplasm | ICD | D35 |
| tumor_dx | benign neoplasm | ICD | D36 |
| tumor_dx | neoplasms of uncertain or unknown behaviour | ICD | D37 |
| tumor_dx | neoplasms of uncertain or unknown behaviour | ICD | D38 |
| tumor_dx | neoplasms of uncertain or unknown behaviour | ICD | D39 |
| tumor_dx | neoplasms of uncertain or unknown behaviour | ICD | D40 |
| tumor_dx | neoplasms of uncertain or unknown behaviour | ICD | D41 |
| tumor_dx | neoplasms of uncertain or unknown behaviour | ICD | D42 |
| tumor_dx | neoplasms of uncertain or unknown behaviour | ICD | D43 |
| tumor_dx | neoplasms of uncertain or unknown behaviour | ICD | D44 |
| tumor_dx | neoplasms of uncertain or unknown behaviour | ICD | D45 |
| tumor_dx | neoplasms of uncertain or unknown behaviour | ICD | D46 |
| tumor_dx | neoplasms of uncertain or unknown behaviour | ICD | D47 |
| tumor_dx | neoplasms of uncertain or unknown behaviour | ICD | D48 |
| laboratory | urea, phosphate and /or calcium clearance | EBM | 32197 |
| laboratory | Calcium | EBM | 32082 |
| laboratory | calcium in urine, atomic absorption spectrometry (AAS) | EBM | 32265 |
| laboratory | Inorganic phosphorus / urinary phosphate exam | EBM | 32086 |
| laboratory | quantitative chemical or physical determination | EBM | 32262 |
| laboratory | electrophoretic separation of human proteins | EBM | 32469 |
| laboratory | alkaline phosphate | EBM | 32068 |
| laboratory | gamma GT | EBM | 32071 |
| laboratory | creatine | EBM | 32236 |
| laboratory | CRP | EBM | 32128 |
| laboratory | TSH | EBM | 32101 |
| laboratory | osteocalcin | EBM | 32414 |
| laboratory | parathyroid hormone intact (PTH) | EBM | 32411 |
| laboratory | DPD | EBM | 32308 |
| laboratory | FGF 23 | EBM | 32416 |
| laboratory | 1,25 OH-Vit D3 (Calcitrol, active Vit D) | EBM | 32421 |
| laboratory | 25 OH Vit D | EBM | 32413 |
| laboratory | additional flat rate kidney function diagnostics | EBM | 17340 |
| scintigram | scintigram | OPS | 370 |
| SPECT | single photon emission computed tomography | OPS | 372 |
| SPECT | single photon emission computed tomography with computed tomography (SPECT/CT) | OPS | 373 |
| PET/ PET/CT | positron emission tomography (PET) with full-ring scanner | OPS | 374 |
| PET/ PET/CT | PET/CT | OPS | 375 |
| EBT | electron beam tomography (EBT) | OPS | 326 |
| PET/ PET/CT | diagnostic positron emission tomography (PET), diagnostic positron emission tomography with computed tomography (PET / CT) | EBM | 347 |
| PET/ PET/CT | F-18 fluorodeoxyglucose PET / CT of the body trunk | EBM | 34701 |
| PET/ PET/CT | F-18 fluorodeoxyglucose PET / CT of the body trunk | EBM | 34702 |
| PET/ PET/CT | F-18 fluorodeoxyglucose PET / CT parts of the body trunk | EBM | 34703 |
| SPECT | SPECT | EBM | 17362 |
| SPECT | SPECT | EBM | 17363 |
| scintigram | whole body scintigram | EBM | 17311 |
| scintigram | partial body scintigram | EBM | 17310 |
| Tumor_removal | excision and destruction of diseased intracranial tissue | OPS | 5015 |
| Tumor_removal | excision and destruction of diseased tissue of the cranial bones | OPS | 5016 |
| Tumor_removal | excision and destruction of diseased tissue of the spinal cord and spinal meninges | OPS | 5035 |
| Tumor_removal | excision and resection of diseased bone tissue | OPS | 5782 |
| Tumor_removal | other surgeries on the bone (thermoablation) | OPS | 5789 |
| Tumor_removal | other surgeries on the bone: destruction, by radiofrequency thermoablation, percutaneous | OPS | 57896 |
| Tumor_removal | destruction of bone tissue by radiofrequency ablation, percutaneous | OPS | 5839h |
| Tumor_removal | implantation or replacement of a tumour endoprosthesis | OPS | 5829c |
| Tumor_removal | incision of diseased bone and joint tissue of the spine | OPS | 5830 |
| Tumor_removal | excision of diseased bone and joint tissue of the spine | OPS | 5832 |
| Tumor_removal | excision of tendons and muscles, epifascial (incl. extirpation of soft tissue tumours) | OPS | 58528 |
| Tumor_removal | excision of tendons and muscles, subfascial (incl. extirpation of soft tissue tumours) | OPS | 58529 |
| Tumor_removal | percutaneous destruction of soft tissues by thermal ablation | OPS | 58593 |
| Tumor_removal | percutaneous destruction of soft tissues by thermal ablation - chest wall and back | OPS | 585935 |
| Tumor_removal | percutaneous destruction of soft tissues by thermal ablation - Thigh and knee | OPS | 585938 |
| Tumor_removal | radiotherapy | OPS | 852 |
| Tumor_removal | therapy of benign diseases by means of soft-jet or orthovoltage therapy | EBM | 2531 |
| Tumor_removal | irradiation with linear accelerator for benign disease | EBM | 25316 |
| Tumor_removal | brachytherapy | EBM | 2533 |
| Gene_testing | basic diagnostics for unclear symptom complex in newborns, infants and children | OPS | 1944 |
| Gene_testing | with extended molecular genetic diagnostics | OPS | 19441 |
| Gene_testing | human genetics fee schedule items | EBM | 11 |
| Gene_expression | carrying out gene mutation analyses and gene expression analyses in solid malignant neoplasms | OPS | 1992 |
| Gene_expression | carrying out gene mutation analyses and gene expression analyses in solid malignant neoplasms | EBM | 19501 |
| Gene_expression | carrying out gene mutation analyses and gene expression analyses in solid malignant neoplasms | EBM | 19502 |
| Gene_expression | carrying out gene mutation analyses and gene expression analyses in solid malignant neoplasms | EBM | 19506 |
| Gene_expression | in vitro diagnostics of tumour genetic alterations | EBM | 194 |
| Gene_expression | FGF23 tumor staining | EBM | 19320 |
| medication | alfacalcidol | ATC | A11CC03 |
| medication | calcitriol | ATC | A11CC04 |
| medication | other urinary calculus dissolving agents, combinations (Reducto) | ATC | G04BC50 |
| medication | calcium | ATC | A12AA |
| Medication | burosumab | ATC | M05BX05 |
| Medication | burosumab | OPS | 600b4 |
